# Study of Vitamin-D Deficiency among Pregnant Women in their First Trimester Visiting a Tertiary Care Hospital: A Descriptive Cross-sectional Study

**DOI:** 10.31729/jnma.6235

**Published:** 2021-07-31

**Authors:** Dipty Shrestha, Rachana Saha, Chandrima Karki, Shilpi Mahato

**Affiliations:** 1 Department of Obstetrics and Gynaecology, Kathmandu Medical College and Teaching Hospital, Sinamangal, Kathmandu, Nepal

**Keywords:** *first trimester*, *pregnancy*, *prevalence*, *Vitamin-D deficiency*

## Abstract

**Introduction:**

Vitamin-D deficient pregnant women are more likely to have pregnancy complications like pre-eclampsia, intrauterine growth restriction, diabetes, preterm birth, etc. Associated factors include ethnicity, skin coverall, use of sun protection, overweight, vitamin-D intake, and smoking. The objective of this study is to determine the prevalence of Vitamin-D deficiency among pregnant women in a tertiary level hospital.

**Methods:**

This study descriptive cross-sectional study was conducted at a tertiary care hospital from September 15th 2020 to November 15th 2020 where the pregnant women visiting for an antenatal checkup in the first trimester were enrolled. Ethical clearance was taken from Institutional Review Committee (reference no. 1808202003). A convenience sampling method was used. All the data were entered in the Statistical Package of the Social Sciences version 20.0 and analyzed. Point estimate at 90% Confidence Interval was calculated along with frequency and percentage for binary data.

**Results:**

Among a total of 47 cases, the vitamin-D deficiency was seen in 21 (44.6%) (32.67-56.52 at 90% Confidence Interval).

**Conclusions:**

The prevalence of vitamin-D deficiency was similar to other studies done in similar settings.

## INTRODUCTION

Vitamin-D deficiency (VDD) is identified as a public health problem in many countries, and pregnant women been identified as a high risk group, among whom the prevalence of VDD ranges between 20-40%.^1^ Vitamin-D and calcium requirements during pregnancy are higher than the normal recommended dose.^2^ Several studies have reported the relationship between maternal VDD and adverse maternal and fetal outcomes, including gestational diabetes, pre-eclampsia, preterm labor, low birth weight, and caesarean section.^3-6^ Maternal VDD is associated with various problems in their babies such as preterm delivery, low birth weight, neonatal hypoglycemia etc. that can be associated with neonatal deaths.^7^

There is still a lack of consensus regarding the vitamin-D supplementation during pregnancy and is necessary to identify the prevalence of VDD in pregnancy and its associated factors.

Thus, the aim of the study is to determine the prevalence of VDD among pregnant women in their first trimester visiting Kathmandu Medical College.

## METHODS

This was a descriptive study at Kathmandu Medical College and Teaching Hospital (KMCTH) conducted from 15th September 2020 to November 15th 2020. Ethical clearance (reference no.1808202003) was taken from the Institutional Review Committee of the same institution. The study was done among pregnant women in the first trimester coming for the antenatal visit at KMCTH. All the pregnant women in the first trimester visiting KMCTH for antenatal visits were included in the study. The exclusion criteria were pregnant women with chronic hypertension, known cases of diabetes mellitus, previous history of hypertension, gestational diabetes mellitus, or intrauterine growth restriction. Convenience sampling was done and the sample size was calculated as,

n = Z^2^ × p × q / e^2^

  = (1.645)^2^ × (0.2) × (1-0.2) / (0.01)^2^

  = 43

Where,

n = minimum required sample size
Z = 1.645 at 90% Confidence Interval
p = prevalence of vitamin-D deficiency among pregnant women, 20%^1^
q = 1-p
e = margin of error, 10%

The required sample size was 43. Adding 10% nonresponse rate, the sample size of 47 was required for the study. Hence, we enrolled 47 participants.

All the cases enrolled in the study were explained about the study and an informed consent was taken. Demographic and clinical data was collected in the Outpatient Department (OPD) using a standardized questionnaire. The questionnaire included demographic profile, general history, past and present obstetrics history, history of past illness, family history, drug history. About 5ml of venous blood was drawn and sent to the laboratory to assess the vitamin-D level. The vitamin-D level was estimated by the Beckman Coulter UniCel DXI immunoassay system. VDD was categorized according to the Institute of Medicine, Food and Nutrition Board. Dietary Reference Intakes for Calcium and Vitamin-D. Washington DC as deficiency - <12ng/dl, insufficient- 12-20ng/dl, sufficient- >20ng/dl, and adverse event- >50ng/dl. Pregnant women fulfilling the criteria were enrolled in the OPD, six times a week.

The data were entered in the Statistical Package for the Social Sciences (SPSS) version 20 for analysis. Point estimate at 90% CI was calculated along with frequency and proportion for binary data.

## RESULTS

**Figure 1:**
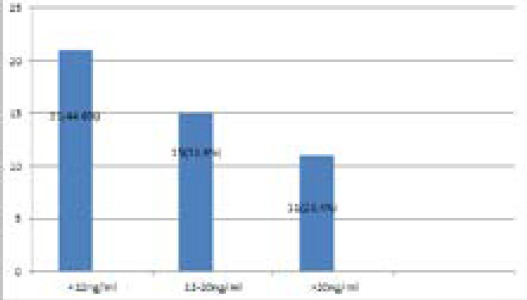
Vitamin-D deficiency status.

Among the participants, deficiency of vitamin D was seen in 21 (44.6%) (32.67-56.52 at 90% CI). Of the total, insufficiency was seen in 15 (31.9%) and 11 (23.4%) of them had sufficient status (Figure 1).

**Figure 2:**
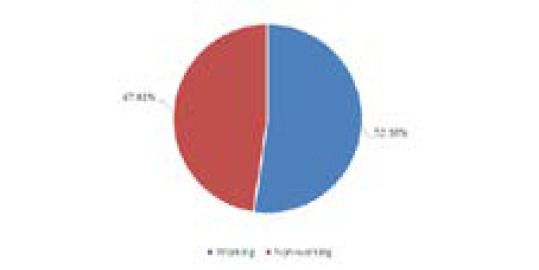
Working status among pregnant women with Vitamin-D deficiency.

Among the total 21 women with VDD, 11 (52.38%) of them had working status whereas 10 (47.61%) of them were non-working (Figure 2).

Most of the pregnant women enrolled were nonvegetarian (Table 1).

**Table 1:** Dietary Status among pregnant women with Vitamin-D deficiency.

Diet	Vegetarian n (%)	Non - vegetarian n (%)	Total n (%)
	3 (14.28)	18 (85.71)	21 (100)

**Figure 3:**
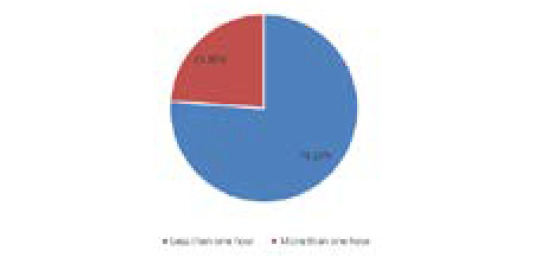
Sun exposure status.

Out ofthe total 21 pregnant womenwith VDD,maximum of them had sun exposure of less than one hour daily 16 (76.19%) and 5 (23.80%) of them had sun exposure of more than one hour daily (Figure 3).

**Figure 4:**
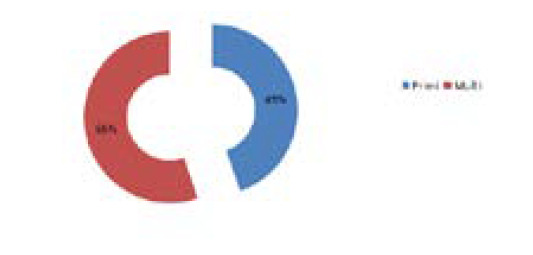
Parity of first trimester women enrolled.

Among the total 47 pregnant women enrolled, 21 (44.68%) of them were primigrávida and 26 (55.3%) were multigravida (Figure 4).

## DISCUSSION

Vitamin-D is an essential element for a pregnant woman as well as neonates. VDD during pregnancy is known to be associated with various adverse neonatal outcomes and also pregnancy complications such as Pre-eclampsia, Gestational Diabetes Mellitus, Preterm labour etc. The World Health Organization (WHO) has recommended one to two grams of elemental calcium during pregnancy and childbirth but not a regular vitamin-D supplementation during normal pregnancy.^8-11^ However, the Royal College of Obstetricians and Gynecologists (RCOG) of the United Kingdom and the American College of Obstetricians and Gynecologists (ACOG) has recommended at least 400 IU of vitamin-D3 supplementation during pregnancy.^12,13^ In this study, out of the total 47 cases, 31 of them were in the age group of 18-30 years and primigravida were 44.68% and multigravida were 55.31%. In a similar study by Shrestha D, et al. in Bhaktapur, the mean age of mothers was 26.7 years with a minimum of 18 years and the maximum of 38 years.^2^ In another study by Arif Sabta Aji E, et al. in Indonesia the mean age was 29.77±5.68yearswith most subjects in the >30-year age group 45.30%. Approximately 75% of them were multiparous and 25% were nulliparous.^14^ In a study by Gupta S, et al., out of the total 137 women with VDD (less than 20ng/ml), 31.4% were multigravida and 68.6% were primigravida, out of the total 24 women with severe VDD (less than 4ng/ml), 20.8% were multigravida and 79.2% were primigravida and out of the total 28 women with Vitamin-D insufficiency (between 20-30ng/ml), 32.1% were multigravida and 67.9% were primigravida.^15^

In this study of mine, among the 21 cases with VDD 52.38% of them were working and 47.61% were nonworking; 14.28% were vegetarian and 85.71% of them were non-vegetarian; only 1 of them had history of intake of vitamin-D and calcium intake; 76.19% had sun exposure of less than 1 hr daily and 23.80% of them had sun exposure more than 1 hour daily. The exposure duration was 60 minutes; 47.80% of the subjects had less than an hour of sun exposure during the day while 52.20% had more than 60 minutes.^14^ The occupation status of the subjects was as follows: 75.40% worked indoors and 24.60% worked outdoors.^14^ Contradictory to this study in a study by Gupta S, et al. out of the total 137 women with VDD (less than 20ng/ml), 26 (19.0%) were non-vegetarian and 111 (81.0%) were vegetarian, out of the total 24 women with severe VDD (less than 4ng/ml), 5 (20.8%) were non-vegetarian and 19 (79.2%) were non-vegetarian and out of the total 28 women with Vitamin-D insufficiency (between 20-30 ng/ml), 4 (14.3%) were non-vegetarian and 24 (85.7%) were vegetarian.^15^

Regarding the Vitamin-D level in pregnant women in this study where the Vitamin-D level was categorized according to IOM; VDD, Vitamin-D insufficiency and sufficient was seen in 44.68%, 31.91%, and 23.40% pregnant women respectively. A study by Shrestha D, et al. in Bhaktapur revealed that the prevalence VDD (<20ng/ml) and insufficient Vitamin-D level (20-30 ng/ml) among pregnant women at the time of delivery were 81% (64/79) and 11.39% (9/79) respectively.^2^ In a similar study to mine by Arif Sabta Aji E, et al. in Indonesia; 47% were Vitamin-D deficit with serum level lower than 12ng/ml; 36.20% were vitamin-D insufficient ( concentration levels of serum between 12-19ng/ml); and 17.20% had sufficient vitamin-D status (concentration levels of serum between 12-19ng/ml).^14^ Among those 21 pregnant women with Vitamin-D level <12ng/ml in this study;16 of them had sun exposure less than hour daily while five of them had sun exposure more than one hour daily. Similarly, 11 out of the 21 pregnant women with Vitamin-D level <12ng/ml of them had working status whereas 10 were non-working and all the four pregnant women who were vegetarian had vitamin level less than 12ng/ml.

The limitation of this study is that this is a small-scale study and due to the ongoing coronavirus disease pandemic, there was a decrease in the number of the antenatal patients. Other studies with a larger sample size and those that look into maternal and fetal outcomes, and those that include pregnant women of all three trimesters must be done in the future.

## CONCLUSIONS

The prevalence of VDD was similar to other studies done in similar settings.
